# MRI-based deep-learning radiomics nomogram for differentiating FIGO 2023 stage I from stage II–IV endometrial cancer: a multicenter study

**DOI:** 10.1186/s13244-026-02428-9

**Published:** 2026-11-06

**Authors:** Xiao Chu, Liting Gao, Xinyi Wang, Tao Quan, Yuxin Ding, Ying Chen, Genji Bai, Mingxiang Wei, Shuangqing Chen

**Affiliations:** 1 Department of Radiology, The Affiliated Suzhou Hospital of Nanjing Medical University, Suzhou Municipal Hospital, Gusu School, Nanjing Medical University, Suzhou, Jiangsu China; 2 Department of Radiology, The First Affiliated Hospital of Soochow University, Suzhou, Jiangsu China; 3 Department of Radiology, The Affiliated Huaian No. 1 People’s Hospital of Nanjing Medical University, Huaian, Jiangsu China

**Keywords:** Magnetic resonance imaging, Radiomics, Deep learning, Nomogram, Endometrial cancer

## Abstract

**Objectives:**

Preoperative identification of FIGO 2023 stage I endometrial cancer (EC) is important for treatment planning. We aimed to develop and validate a deep-learning radiomics nomogram (DLRN) based on multiparametric MRI to distinguish stage I EC from stage II–IV disease.

**Materials and methods:**

This study retrospectively enrolled patients from three centers. Patients from Centers 1 and 2 were randomly split into a training set (*n* = 616), and an internal test set (*n* = 158), and Center 3 served as an external test set (*n* = 232). The DLRN integrated deep-learning features, intratumoral and peritumoral radiomics features, and clinical characteristics. The DLRN was compared with clinical, radiomics, and deep-learning models. Discrimination was evaluated using the area under the receiver operating characteristic curve (AUC), with AUCs compared using DeLong’s test.

**Results:**

A total of 1006 patients (mean age, 56.5 ± 9.7 years) were analyzed. In the internal test set, the DLRN achieved an AUC of 0.852, higher than the clinical (0.673; *p* = 0.001), original radiomics (0.765; *p* = 0.030), and combined radiomics (0.793; *p* = 0.050) models. In the external test set, the DLRN remained robust (AUC = 0.829) and outperformed the clinical, original radiomics, and combined radiomics models (AUC = 0.680, 0.681, and 0.743; all *p* < 0.005). With DLRN assistance, radiologists showed improved diagnostic performance.

**Conclusion:**

The MRI-based DLRN showed robust discrimination between FIGO 2023 stage I and stage II–IV EC across test sets, supporting its potential for preoperative risk stratification and decision support.

**Key Points:**

***Question*** The introduction of the FIGO 2023 staging system has made the differentiation between stages more challenging, highlighting the need for better tools.

***Findings*** A new MRI-based deep-learning radiomics nomogram (DLRN) accurately identifies high-risk patients, significantly outperforming traditional and individual clinical prediction methods.

***Critical relevance statement*** This tool helps doctors make better preoperative treatment plans, potentially improving patient outcomes in endometrial cancer.

**Graphical Abstract:**

**Figure:**
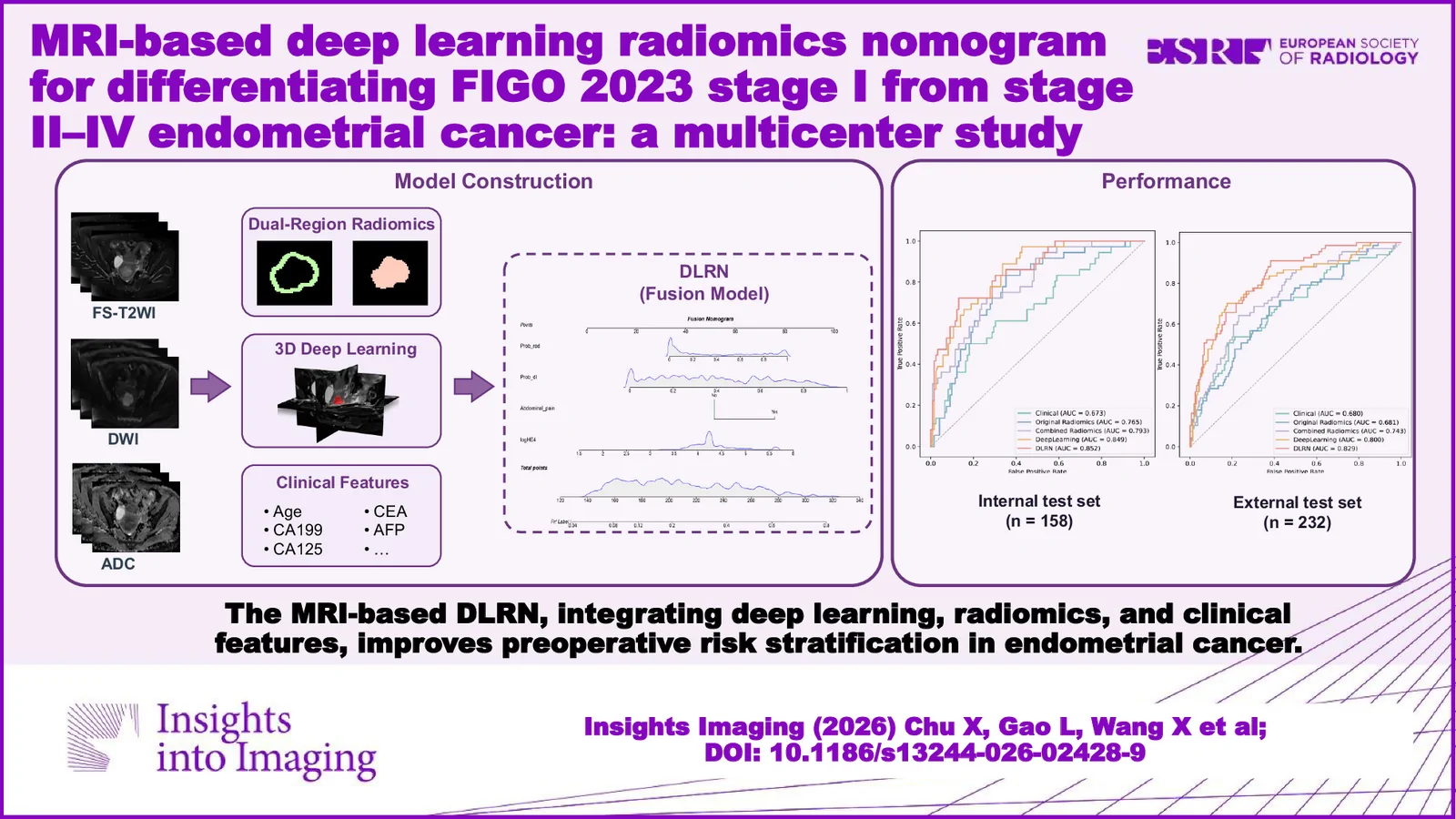

## Introduction

Endometrial cancer (EC) is one of the most common malignancies in women, with both global incidence and EC-related mortality rising in recent years [1]. Notably, an increasing proportion of cases are diagnosed in younger women [2]. Because many of these patients have not yet completed childbearing [3], accurate pre-treatment risk stratification is critical to support fertility-sparing options without compromising oncologic outcomes.

The release of the International Federation of Gynecology and Obstetrics (FIGO) 2023 staging system has fundamentally redefined the requirements for preoperative assessment, shifting toward a more granular risk-stratification model [4]. The revised criteria place greater prognostic weight on specific histopathological and invasive markers—such as the depth of myometrial invasion, cervical stromal involvement, and lymphovascular space invasion [5]. In this context, reliably differentiating FIGO 2023 stage I from stage II–IV disease is essential: patients with stage I disease may be candidates for fertility-sparing or less radical surgical approaches, whereas stage II–IV disease typically warrants more comprehensive surgical staging and intensified adjuvant treatment [2, 6–8]. Therefore, highly accurate preoperative staging is needed to support tailored surgical planning and adjuvant management.

MRI is a key modality for preoperative assessment of EC [9, 10], providing critical insights into tumor extension and lymph node status [11–13]. However, as FIGO 2023 increasingly integrates pathological and molecular indicators [1], identifying subtle imaging correlates remains challenging. Such assessments often rely heavily on reader experience, which may compromise consistency and reproducibility. Therefore, developing an objective, reproducible, preoperative decision-support tool is clinically important [14].

In recent years, the rapid development of radiomics and deep learning has opened new avenues for preoperative evaluation of EC [15–18]. Radiomics enables high-throughput extraction of quantitative features from MRI and other imaging modalities to characterize tumor heterogeneity [19, 20]. Few studies have integrated radiomics and deep learning to discriminate between FIGO 2023 stage I and stage II–IV EC preoperatively. The peritumoral microenvironment is closely linked to invasion and dissemination, suggesting that combining intratumoral and peritumoral features may improve risk stratification [21, 22].

Accordingly, we conducted a multicenter retrospective study to develop and validate an MRI-based deep-learning radiomics nomogram (DLRN) to preoperatively distinguish FIGO 2023 stage I from stage II–IV EC. We also explored whether combining deep-learning features with intratumoral and peritumoral features could provide additional diagnostic and risk-stratification benefits to support clinical decision-making.

## Materials and methods

This retrospective study was approved by the Affiliated Suzhou Hospital of Nanjing Medical University, Suzhou Municipal Hospital (protocol number: K-2025-322-K01), and the requirement for written informed consent was waived. The study was conducted in accordance with the Declaration of Helsinki.

### Patients

**Fig. 1:**
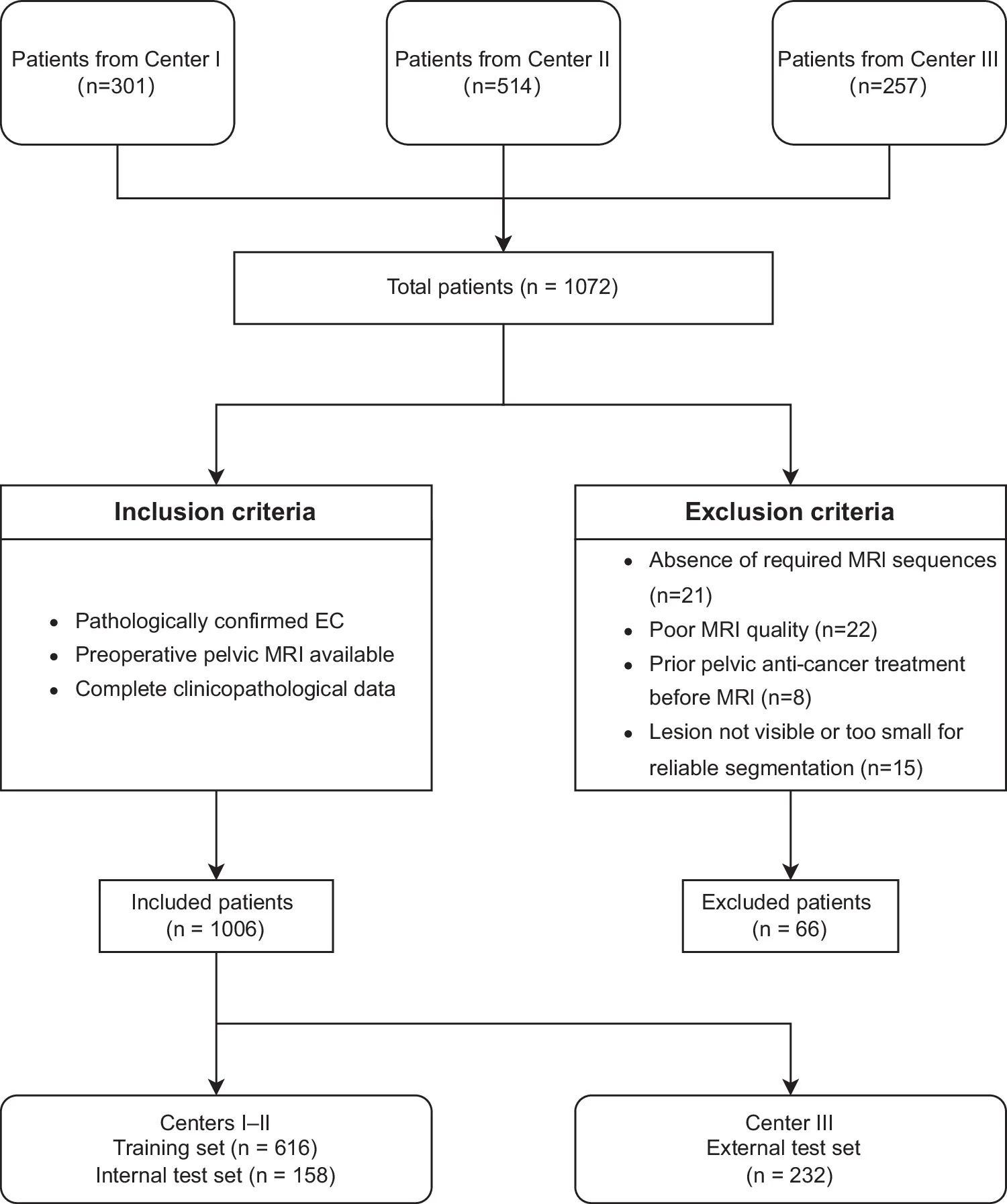
Flowchart of patient inclusion, exclusion, and set allocation. EC, endometrial cancer; MRI, magnetic resonance imaging

We retrospectively reviewed consecutive patients who underwent surgery for EC at three centers between 2013 and 2023 and had preoperative pelvic MRI. Inclusion criteria were: (1) pathologically confirmed EC; (2) availability of preoperative pelvic MRI with the required sequences; and (3) complete clinicopathological data. Exclusion criteria were: missing required MRI sequences (*n* = 21), poor MRI image quality (*n* = 22), prior pelvic anti-cancer treatment before MRI (*n* = 8), and lesions not visible or too small for reliable segmentation (*n* = 15). A total of 1072 patients were screened; 66 were excluded, leaving 1006 patients for analysis. Patients from Centers 1 and 2 were pooled and randomly split into a training set (*n* = 616) and an internal test set (*n* = 158), while patients from Center 3 served as an independent external test set (*n* = 232) to evaluate the model’s generalizability. The patient selection process is summarized in Fig. 1. MRI, surgery, and relevant clinical/laboratory assessments were completed within 15 days.

Baseline characteristics were available from the clinical record system, including age, menopausal status, abnormal uterine bleeding, abdominal symptoms, alpha-fetoprotein (AFP), serum cancer antigen 19-9 (CA19-9), cancer antigen 125 (CA125), carcinoembryonic antigen (CEA), human epididymal protein 4 (HE4), and histopathological subtype. All patients with EC underwent staging surgery and were classified according to the latest FIGO 2023 staging system (detailed in Supplementary Materials Section S1).

### Image acquisition and preprocessing

**Fig. 2:**
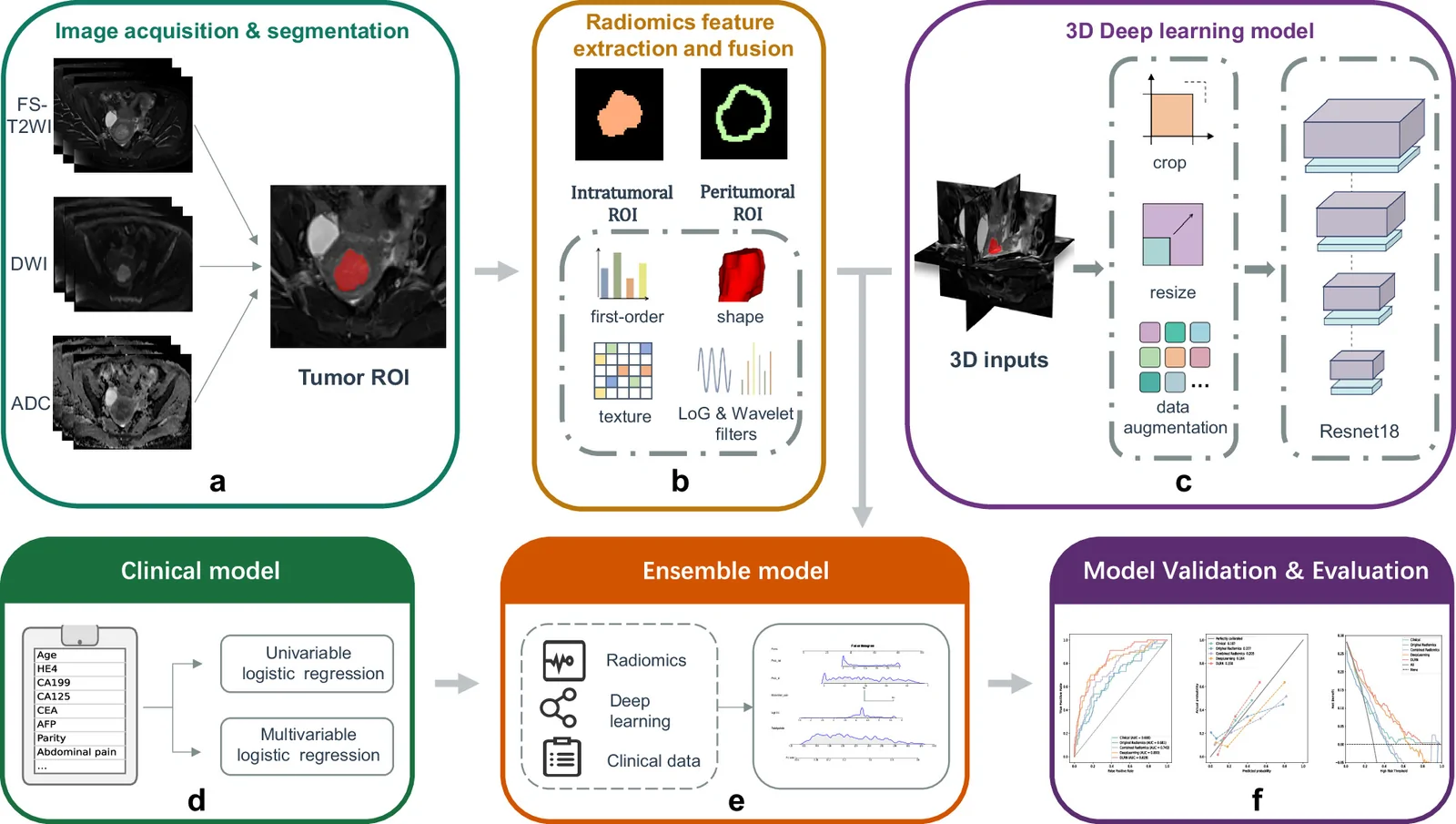
Schematic overview of the study workflow. **a** Image acquisition and intratumoral ROI segmentation on multiparametric MRI (FS-T2WI, DWI, and ADC). **b** Radiomics feature extraction from intratumoral and peritumoral ROIs and construction of the combined radiomics model. **c** Development of the 3D deep-learning model. **d** Development of the clinical model. **e** Construction of the ensemble model (DLRN) integrating radiomics, deep learning, and clinical characteristics. **f** Model validation and performance evaluation. ROI, region of interest; FS-T2WI, fat-suppressed T2-weighted imaging; DWI, diffusion-weighted imaging; ADC, apparent diffusion coefficient; DLRN, deep-learning radiomics nomogram. AFP, CA125, CA19-9, CEA, and HE4 are serum biomarkers

An overview of the study workflow is shown in Fig. 2. Center information and MRI parameters are detailed in Supplementary Materials Section S2 and Table S1 and S2. Using fat-suppressed T2-weighted imaging (FS-T2WI) as the reference, diffusion-weighted imaging (DWI) and apparent diffusion coefficient (ADC) images were registered to the FS-T2WI images. If a patient underwent multiple preoperative MRI examinations, the scan closest to surgery was selected for analysis. All images underwent N4 bias field correction. Given voxel-spacing differences across scanners and imaging protocols, all sequences were resampled to isotropic voxels of 1 × 1 × 1 mm³ using B-spline interpolation, and image intensities were normalized to the range of 0–1.

### Image segmentation

Tumors were manually segmented on each slice of the FS-T2WI images using ITK-SNAP (version 4.2.0; http://www.itksnap.org) to generate the original regions of interest (ROIs). Each original ROI encompassed the entire tumor volume.

All ROIs were delineated by a radiologist (R1) with 10 years of experience in pelvic MRI interpretation and were verified by a senior radiologist (R2) with more than 15 years of experience. Any discrepancies were resolved by consensus. To assess interobserver reproducibility for radiomics analysis, another radiologist (R3) with 30 years of pelvic MRI experience independently delineated ROIs for 30 patients randomly selected from the training set. To evaluate intraobserver reproducibility, R1 repeated segmentation for these 30 cases after a 1-month washout period. Reproducibility between and within observers was quantified using the intraclass correlation coefficient (ICC), with ICCs > 0.80 indicating good reproducibility. To minimize bias, all radiologists involved were trained in the use of segmentation software and were blinded to the patients’ histopathological results.

### Radiomics analysis

**Fig. 3:**
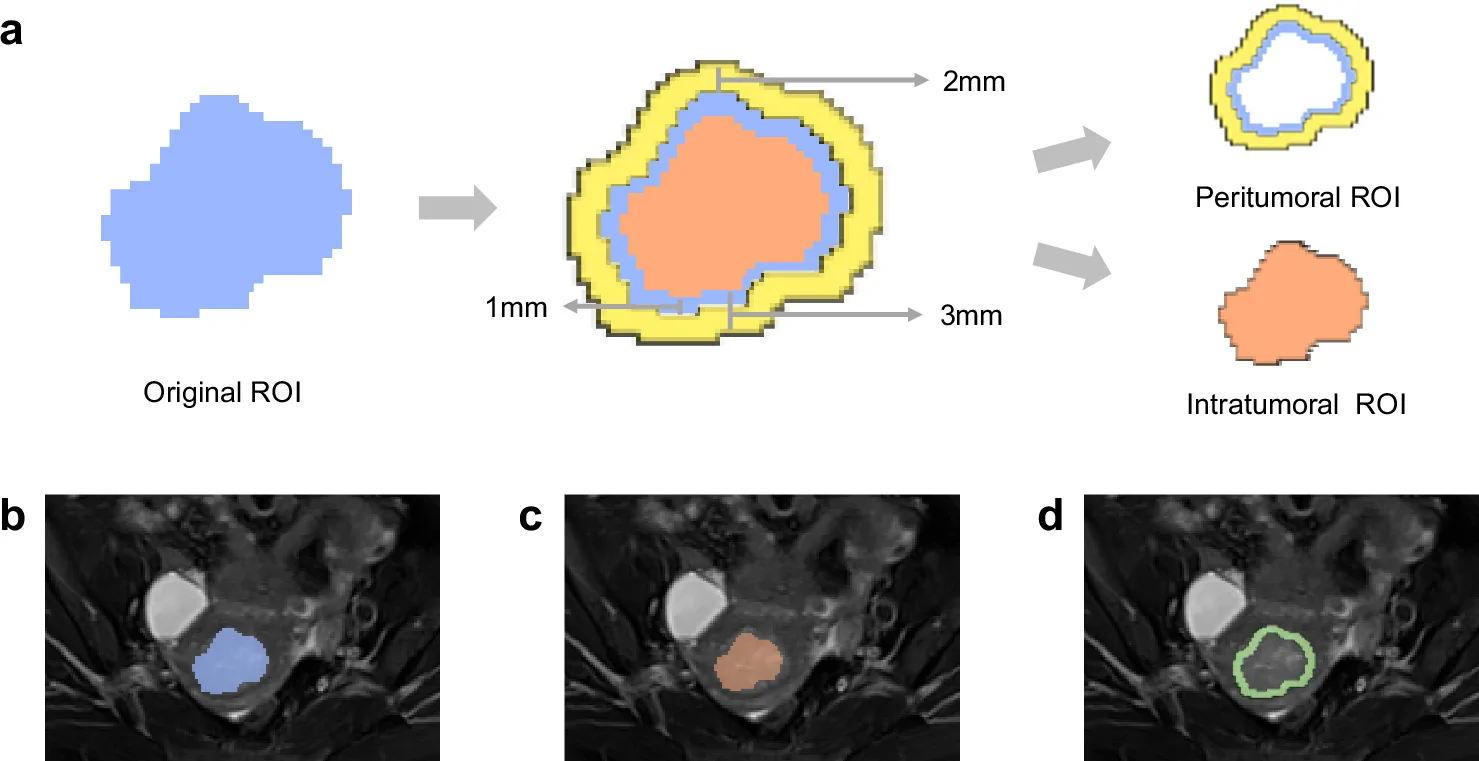
Illustration of intratumoral and peritumoral ROI generation. **a** The original tumor ROI was processed by a 2 mm outward dilation and a 1 mm inward erosion; the peritumoral ROI was defined as the annular boundary ring between these two masks, and the intratumoral ROI was obtained by a 1 mm erosion of the original ROI. **b** Original ROI overlaid on the MR image. **c** Intratumoral ROI overlaid on the MR image. **d** Peritumoral (boundary-ring) ROI overlaid on the MR image. ROI, region of interest

In this study, we constructed a combined radiomics model using an intratumoral–peritumoral radiomics strategy. Intratumoral and peritumoral ROIs were automatically generated from corresponding original ROIs using the SimpleITK open-source package (version 2.4.0). Although a 3 mm peritumoral expansion is commonly used in prior studies [14, 22, 23], to better capture information at the tumor–myometrium interface and to reduce the impact of boundary uncertainty on feature extraction, we defined a 3 mm “peritumoral/boundary ring” as an annular region obtained by taking the set difference between a 2 mm outward dilation and a 1 mm inward erosion of the original ROI (i.e., the region between dilate 2 mm and erode 1 mm). The intratumoral ROI was generated by eroding the original ROI by 1 mm (Fig. 3).

Radiomics features were extracted from intratumoral and peritumoral regions on FS-T2WI, DWI, and ADC sequences using PyRadiomics (version 3.1.0). A total of 6,780 features were obtained from the three sequences and two ROIs. Feature selection was performed using Mann–Whitney *U*-tests, random forest ranking, Spearman correlation, and LASSO (Least Absolute Shrinkage and Selection Operator) with cross-validation. A support vector machine classifier was trained using the selected features, and SMOTEENN was applied to address class imbalance. The model’s performance was evaluated using out-of-fold (OOF) predicted probabilities. Details of feature extraction, selection, and model development are provided in Supplementary Materials Section S3.

### Deep-learning analysis

We developed a three-channel 3D deep-learning classification model based on multiparametric MRI (FS-T2WI, DWI, and ADC). For each modality, a cubic patch centered on the lesion ROI was extracted and resampled to 96 × 96 × 96 voxels. Unlike radiomics analysis, which can be performed on separately defined ROIs, the CNN model needed a standardized input size. Therefore, we used lesion-centered cubic patches as the CNN input rather than separately training intratumoral or peritumoral CNN models. A comparison with enlarged peritumoral patches is provided in Supplementary Table S4. Data augmentation was applied to the training set to improve generalization. A 3D ResNet-18 backbone was used, as it has been commonly used in 3D medical imaging studies and offers a relatively lightweight architecture suitable for our dataset size. The model was optimized using AdamW with binary cross-entropy loss and positive class weight to address class imbalance. Early stopping was implemented, and out-of-fold (OOF) inference was used for evaluation. Model training was performed using PyTorch (version 2.9.1) on an NVIDIA GeForce RTX 4090 GPU. Further details and comparisons with alternative deep-learning backbones are provided in Supplementary Materials Section S4 and Supplementary Table S5.

### Model development and OOF inference

To avoid information leakage, all feature selection and model tuning were performed within the training set only. OOF inference was performed using a single predefined 5-fold (*K* = 5) fold table. Specifically, in each fold, the model was trained on *K* − 1 folds and used to infer the held-out fold; predictions from all folds were concatenated to generate OOF probabilities for the entire training set. Final models were then retrained on the whole training set and used to generate predicted probabilities for the internal test and external test sets.

### Clinical model construction

In the training set, we collected and analyzed clinical variables including age, menopausal status, abnormal uterine bleeding, abdominal pain, parity, and serum levels of CA19-9, CA125, HE4, CEA, and AFP to develop the clinical model. Given the markedly skewed distributions of CA19-9, CA125, and HE4, logarithmic transformation was applied. Univariate logistic regression was then performed for each candidate variable, with *p* < 0.10 used as the screening criterion to identify variables for inclusion in the multivariable logistic regression model [24]. In the multivariable analysis, variables with *p* < 0.05 were considered independent clinical characteristics and were used to construct the clinical model.

### DLRN construction

In this study, we developed a DLRN to integrate multi-source information and provide individualized predicted probabilities. Specifically, the predicted probabilities from the deep-learning model and the combined intratumoral–peritumoral radiomics model, along with the statistically selected independent clinical characteristics, were jointly entered into a binary logistic regression to establish the final fusion model. For clinical interpretability and practical use, the fusion model was further visualized as a nomogram.

### Model evaluation

The discriminative performance of each model was assessed using the AUC; accuracy, sensitivity, and specificity were also calculated. Calibration was evaluated using the Brier score and calibration curves, and decision curve analysis (DCA) was performed to quantify net benefit across a range of threshold probabilities. For interpretability, Shapley Additive exPlanations (SHAP) analysis was used to quantify the contribution of radiomics features, and Gradient-weighted Class Activation Mapping (Grad-CAM) was used to visualize image regions most influential to the deep-learning predictions.

### Statistical analysis

All statistical analyses were conducted using R (version 4.4.2) and Python (version 3.8.2, utilizing the scikit-learn and PyTorch packages). Continuous variables are presented as mean ± standard deviation or median (interquartile range, IQR), depending on their distribution. Categorical variables are expressed as counts (percentages). For between-group comparisons, continuous variables were analyzed using Student’s t-test or the Mann–Whitney *U*-test, as appropriate. In contrast, categorical variables were compared using the chi-square test or Fisher’s exact test, depending on the expected cell counts. Model discriminative performance was evaluated using AUC. The 95% confidence intervals (CIs) of the AUCs were calculated via the DeLong method. Pairwise comparisons of AUCs between different models were performed using the paired DeLong test, conducted separately in the internal test set and the external test set. All statistical tests were two-sided, and *p*-values < 0.05 were considered statistically significant.

## Results

### Clinical characteristics

A total of 1006 patients were enrolled, including 740 patients with stage I EC (mean age, 55.3 ± 9.17 years) and 266 patients with stage II–IV EC (mean age, 59.5 ± 10.1 years). Among patients with stage II–IV EC, 172 (64.7%) were stage II, 82 (30.8%) were stage III, and 12 (4.5%) were stage IV. All patients were divided into a training set (*n* = 616; mean age, 56.42 ± 9.59 years), an internal test set (*n* = 158; mean age, 54.28 ± 9.39 years), and an external test set (*n* = 232; mean age, 56.22 ± 10.58 years). The demographic and clinical characteristics of the patients are summarized in Table 1.

**Table 1:** The demographic and clinical characteristics of the patients

Characteristic	Training set			Internal test set			External test set		
	I EC *N* = 453	II-IV EC *N* = 163	*p-*value	I EC *N* = 122	II-IV EC *N* = 36	*p*-value	I EC *N* = 165	II-IV EC *N* = 67	*p*-value
Age (years)	55.3 ± 9.2	59.5 ± 10.1	< 0.001	53.6 ± 9.3	56.6 ± 9.4	0.102	56.1 ± 10.6	56.5 ± 10.5	0.810
Menopausal status			0.001			0.071			0.804
Premenopausal	174 (38.7%)	40 (24.7%)		54 (44.6%)	10 (27.8%)		49 (29.7%)	21 (31.3%)	
Postmenopausal	276 (61.3%)	122 (75.3%)		67 (55.4%)	26 (72.2%)		116 (70.3%)	46 (68.7%)	
Abnormal uterine bleeding			0.774			0.077			0.063
No	41 (9.1%)	16 (9.9%)		7 (5.8%)	6 (16.7%)		10 (6.1%)	9 (13.4%)	
Yes	409 (90.9%)	146 (90.1%)		114 (94.2%)	30 (83.3%)		155 (93.9%)	58 (86.6%)	
Abdominal pain			0.007			0.188			0.002
No	414 (92.0%)	137 (84.6%)		105 (86.8%)	28 (77.8%)		135 (81.8%)	42 (62.7%)	
Yes	36 (8.0%)	25 (15.4%)		16 (13.2%)	8 (22.2%)		30 (18.2%)	25 (37.3%)	
Parity			0.969			0.686			0.494
Nulliparous	20 (4.5%)	7 (4.4%)		8 (6.8%)	1 (2.8%)		14 (8.6%)	4 (6.0%)	
Multiparous	424 (95.5%)	151 (95.6%)		109 (93.2%)	35 (97.2%)		148 (91.4%)	63 (94.0%)	
CA19-9 (U/mL)	14.0 (8.0, 25.6)	19.2 (8.2, 44.1)	0.011	11.5 (6.6, 21.4)	17.3 (7.1, 39.1)	0.057	12.3 (5.9, 27.3)	11.3 (6.19, 28.4)	0.940
CA125 (U/mL)	17.8 (12.5, 28.6)	22.4 (13.2, 43.7)	< 0.001	17.4 (12.8, 25.7)	25.2 (14.9, 42.3)	0.009	18.6 (13.0, 28.5)	27.1 (16.7, 59.6)	< 0.001
HE4 (pmol/L)	64.8 (50.4, 89.7)	88.3 (64.3, 143.0)	< 0.001	59.8 (48.4, 86.5)	72.7 (55.2, 126.0)	0.046	52.7 (38.9, 66.2)	62.1 (42.9, 99.6)	0.019
CEA (ng/mL)	1.6 (1.1, 2.3)	1.8 (1.2, 2.6)	0.064	1.5 (1.0, 2.5)	1.8 (1.3, 2.7)	0.107	1.7 (1.2, 2.4)	2.0 (1.5, 3.0)	0.026
AFP (ng/mL)	2.7 (2.0, 3.6)	2.8 (2.1, 3.8)	0.094	2.7 (2.0, 3.2)	2.9 (2.0, 3.6)	0.309	2.5 (2.0, 3.5)	2.6 (2.0, 3.3)	0.831

### Model construction

**Fig. 4:**
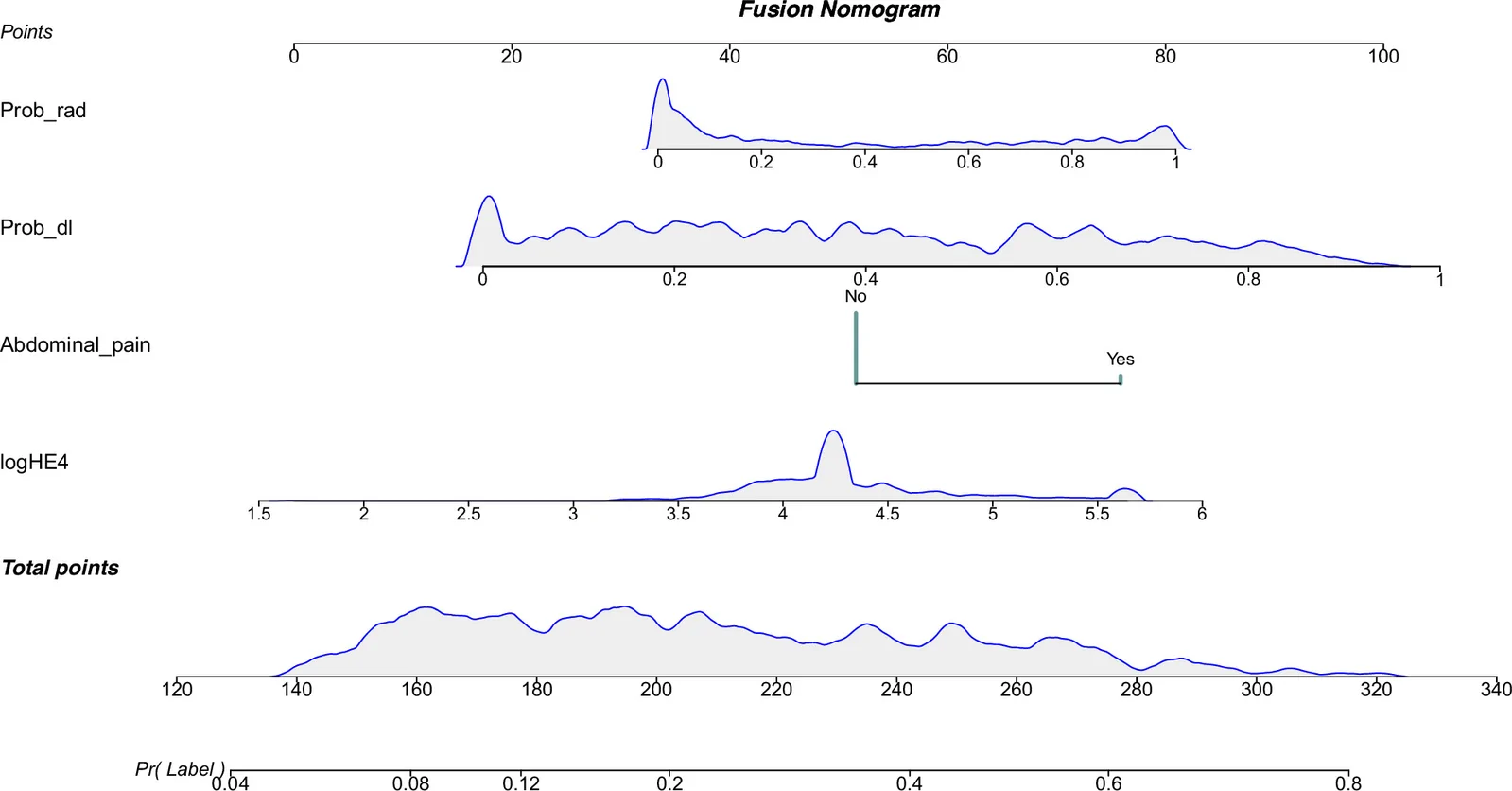
Deep-learning radiomics nomogram (DLRN) for preoperative differentiation between FIGO 2023 stage I and stage II–IV endometrial cancer. Prob_dl, deep-learning probability; Prob_rad, radiomics probability; HE4, human epididymis protein 4

After radiomics feature selection, 12 features from the original ROI were retained to construct the original radiomics model, whereas 11 features from the combined intratumoral and peritumoral feature set were retained to build the combined radiomics model. The SHAP summary plot illustrates the functional relationships between the selected radiomics features and the radiomics model output. logHE4 and abdominal pain were identified as independent clinical characteristics and were used to develop the clinical model. The DLRN integrated the output probabilities from the deep-learning model and the combined radiomics model, together with the independent clinical characteristics, as shown in Fig. 4.

### Model evaluation and comparison

**Fig. 5:**
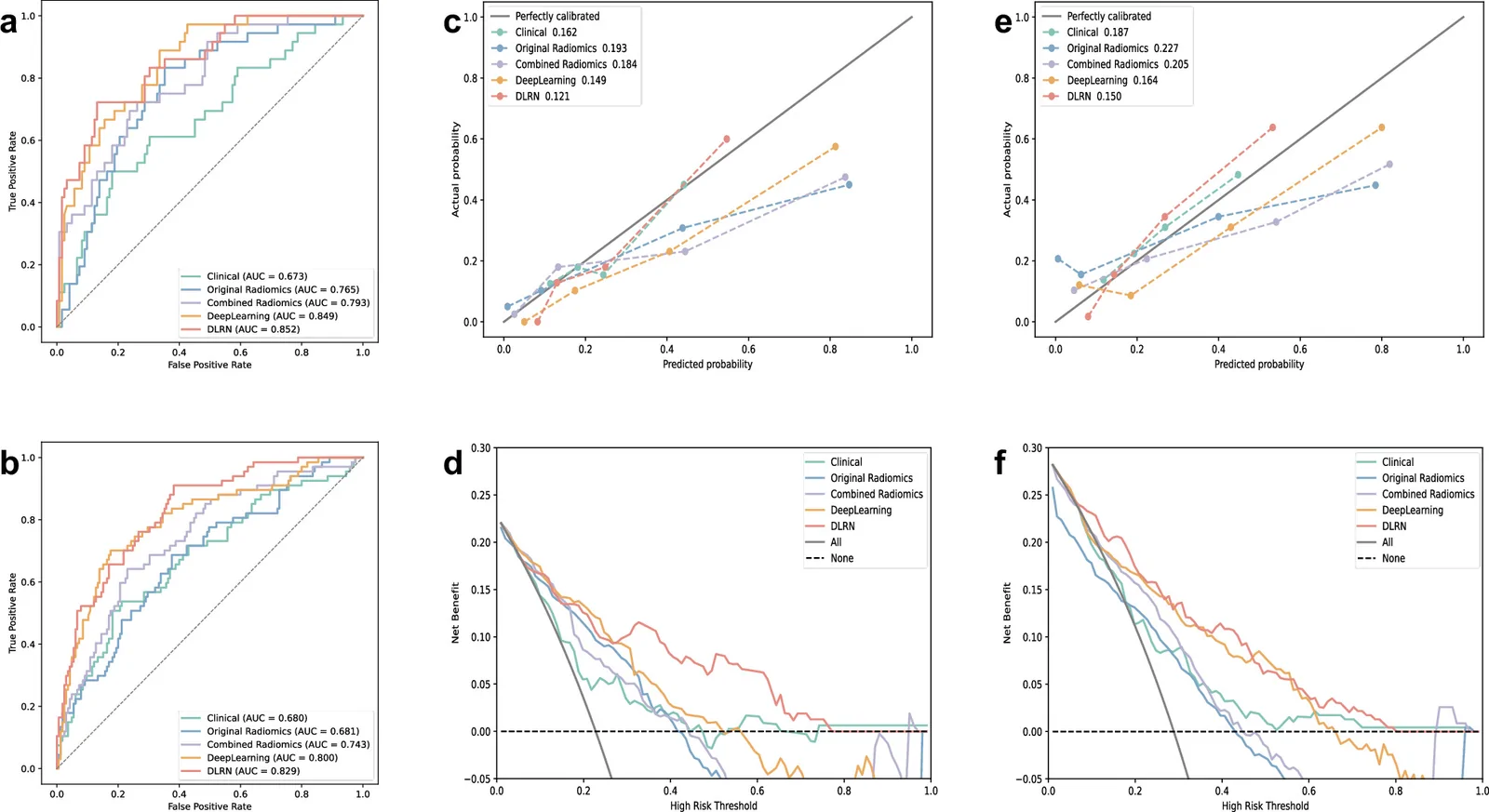
**a**, **b** Receiver operating characteristic (ROC) curves of the five models in the internal test set (**a**) and external test set (**b**), with AUC values shown in the legend. **c**, **d** Calibration curves of the five models in the internal test set (**c**) and external test set (**d**), with the legend reporting Brier scores; the diagonal line indicates perfect calibration. **e**, **f** Decision curve analysis (DCA) of the five models in the internal test set (**e**) and external test set (**f**), showing net benefit across threshold probabilities with “treat-all” and “treat-none” strategies for reference. AUC, area under the receiver operating characteristic curve; DLRN, deep-learning radiomics nomogram; DCA, decision curve analysis

**Fig. 6:**
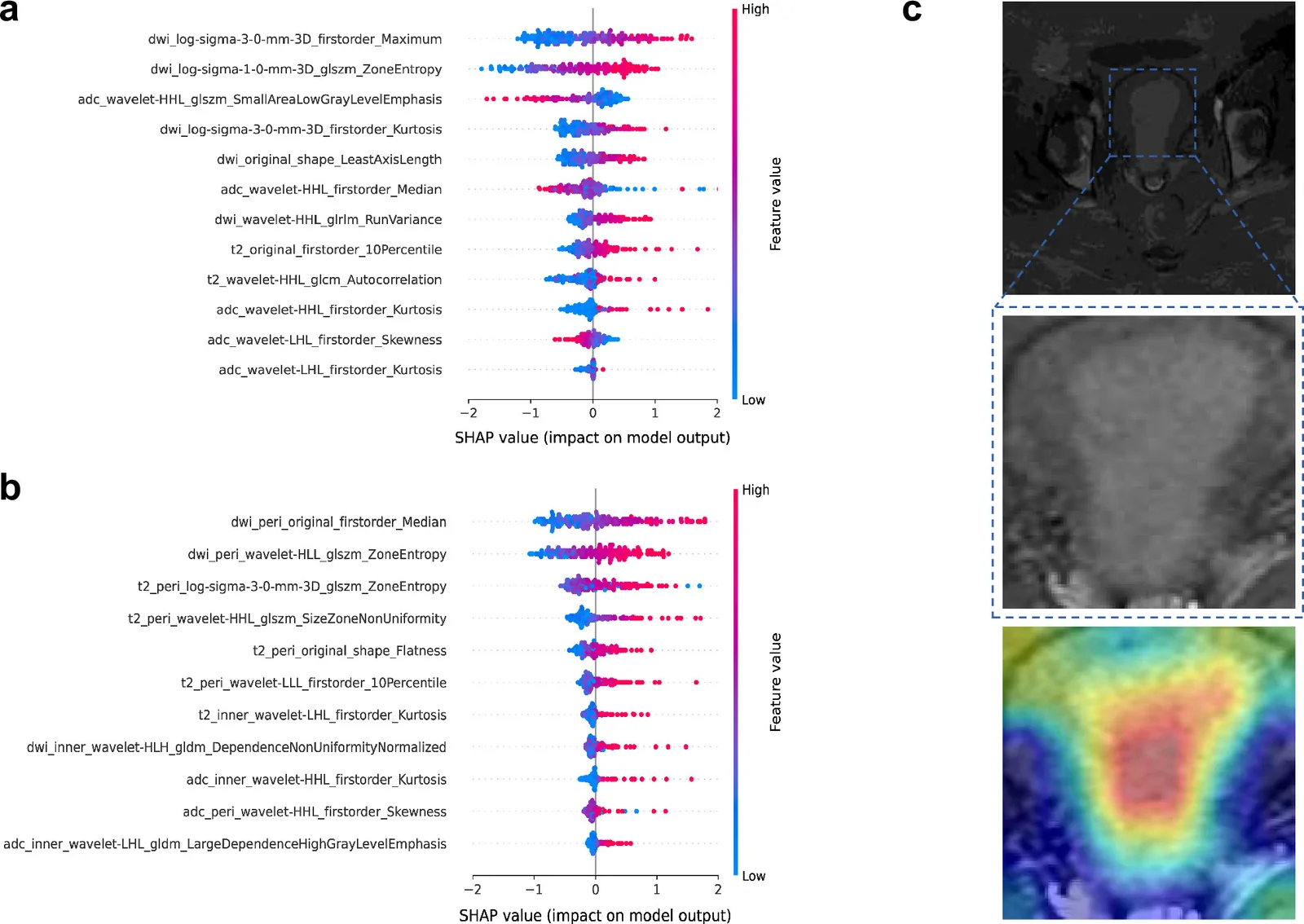
Model interpretability and visualization. **a**, **b** SHAP summary (beeswarm) plots for the original radiomics model (**a**) and the combined radiomics model (**b**), where SHAP values quantify each feature’s contribution to the model output (positive values increase the predicted probability and negative values decrease it); features are ranked by mean absolute SHAP value, and colors indicate feature values from low (blue) to high (red). **c** Example Grad-CAM visualization for a 39-year-old patient with stage III EC, showing the lesion-centered patch and the corresponding attention heatmap (warmer colors indicate greater contribution to the prediction). SHAP, Shapley Additive exPlanations; Grad-CAM, Gradient-weighted Class Activation Mapping; EC, endometrial cancer

The performance of the DLRN and comparator models is presented in Fig. 5 and Table 2. Additional regional radiomics comparisons among intratumoral, peritumoral, and combined intratumoral–peritumoral models are provided in Supplementary Table S3. In the internal test set (*n* = 158), the DLRN achieved the highest AUC of 0.852 (95% CI, 0.783–0.920), significantly outperforming the clinical model (AUC = 0.673; *p* = 0.001) and the original radiomics model (AUC = 0.765; *p* = 0.030), with a borderline significant difference compared with the combined radiomics model (AUC = 0.793, *p* = 0.050). In the external test set (*n* = 232), the DLRN remained the best-performing model, yielding an AUC of 0.829 (95% CI, 0.773–0.884) and significantly outperforming the clinical model, the original radiomics model, and the combined radiomics model (AUC = 0.680/0.681/0.743; *p* < 0.001/ < 0.001/0.005, respectively). However, the DLRN did not significantly outperform the deep-learning model in the external test set by the DeLong test (*p* = 0.183). Decision curve analysis showed that the DLRN provided higher net benefit across most threshold probabilities. Calibration curves also showed better agreement, and the DLRN achieved the lowest Brier scores (internal test: 0.121; external test: 0.150). An example of the model interpretability analysis is shown in Fig. 6. In addition, DLRN assistance improved radiologists’ diagnostic performance in the external test set, as shown in Supplementary Fig. S1.

**Table 2:** Performance of the DLRN and comparator models

Models	Internal test				External test			
	AUC (95% CI)	Acc	Sens	Spec	AUC (95% CI)	Acc	Sens	Spec
Clinical	0.673 (0.570–0.776)	0.734	0.389	0.836	0.680 (0.602–0.758)	0.711	0.522	0.788
Original radiomics	0.765 (0.681–0.848)	0.709	0.722	0.705	0.681 (0.605–0.757)	0.647	0.567	0.679
Combined radiomics	0.793 (0.713–0.873)	0.734	0.722	0.738	0.743 (0.673–0.812)	0.659	0.687	0.648
Deep learning	0.849 (0.785–0.914)	0.747	0.722	0.754	0.800 (0.734–0.866)	0.746	0.716	0.758
DLRN	0.852 (0.783–0.920)	0.741	0.722	0.746	0.829 (0.773–0.884)	0.741	0.716	0.752

## Discussion

In this multicenter study, we developed and validated an MRI-based DLRN to discriminate between FIGO 2023 stage I and stage II–IV EC preoperatively. The integrated model outperformed single-modality models and maintained stable discrimination and calibration across internal and external sets, suggesting potential utility for preoperative risk stratification and decision support. Notably, the model retained consistent performance in an external set, supporting its potential generalizability across centers.

The advantage of the DLRN is likely attributable to its complementary, multi-regional feature representation. The updated FIGO 2023 staging system incorporates a broader spectrum of anatomic and biologic risk factors, increasing the complexity of preoperative staging based on imaging alone. In particular, subtle cervical stromal invasion, early invasive growth patterns, and lymphovascular space invasion—key staging determinants in the FIGO 2023 system—remain challenging to identify on routine MRI, even for experienced radiologists [25]. These ambiguities may partly explain why conventional MRI assessment and single-model approaches show limited performance in multicenter settings.

Prior studies have often focused on individual staging-relevant factors or a single modeling paradigm, and evidence for multicenter preoperative discrimination between FIGO 2023 stage I and stage II–IV disease remains limited [11, 26]. Several MRI-based radiomics nomograms have demonstrated only modest performance, as they lack multi-model integration and were primarily developed under earlier FIGO systems without explicit optimization for the updated FIGO 2023 framework. To address these gaps, our DLRN integrates intratumoral and peritumoral radiomics, deep learning-derived representations, and clinical characteristics within a multicenter design aligned with FIGO 2023. Intratumoral and peritumoral information is highly complementary: the intratumoral ROI reflects intrinsic structural and signal heterogeneity, whereas the peritumoral ROI may serve as an imaging proxy of the tumor–host interface, capturing cues related to invasive margins and microenvironmental remodeling [14, 21, 23]. In the present study, a fixed peritumoral margin was used to provide a reproducible and anatomically interpretable representation of the tumor–myometrium interface while limiting the inclusion of unrelated background tissue. By jointly modeling these multi-regional cues with hierarchical deep-learning features, the DLRN enables more robust characterization of tumor aggressiveness, which may explain its stable performance across internal and external sets, particularly in atypical or borderline cases [27, 28].

Accurate preoperative differentiation between stage I and stage II–IV disease is clinically meaningful because it influences care pathways, surgical extent, nodal assessment strategies, and the anticipated intensity of adjuvant treatment and surveillance [29, 30]. Rather than replacing radiologist assessment, the DLRN is intended to function as a decision-support tool within the preoperative workflow, providing probabilistic evidence in cases with equivocal or discordant findings under the FIGO 2023 framework. Such quantitative risk stratification may assist multidisciplinary teams in refining surgical staging strategies and tailoring postoperative management, thereby improving the consistency of preoperative decision-making in clinical practice.

Interpretability is essential for clinical deployment of AI models. Although radiomics features are explicitly defined mathematical descriptors, it can be difficult to translate a change in feature value into an intuitive clinical explanation; therefore, we used SHAP to summarize how each selected radiomics feature contributed to the final prediction at both the dataset and individual levels. For the deep-learning branch, Grad-CAM was used to generate attention maps, indicating which image regions most influenced the model’s output and allowing clinicians to judge whether the network relied on anatomically and pathologically plausible cues rather than irrelevant patterns. In addition, the DLRN’s fusion step was built on logistic regression, which naturally supports transparent visualization via a nomogram. Together, these outputs provide transparent evidence that can be reviewed alongside clinical judgment.

Several limitations should be acknowledged. First, as a retrospective study conducted across centers within the same region, selection bias may be present, and broader geographic validation is needed. Second, the dataset exhibited class imbalance, with an overrepresentation of stage I cases, which may have influenced model training. To mitigate this issue, we applied resampling strategies, such as SMOTEENN, during training; however, residual bias cannot be eliminated despite stable external performance. Third, for peritumoral radiomics, only a fixed peritumoral margin was evaluated, and the model’s sensitivity to different expansion distances was not assessed. In addition, contrast-enhanced MRI was excluded because of inconsistent availability and protocol heterogeneity; its value should be evaluated in future studies. Finally, molecular subtyping was not incorporated because it was not consistently available across participating centers; future work may integrate imaging phenotypes with molecular pathology to establish a more comprehensive preoperative assessment framework [31].

In conclusion, the DLRN—integrating deep learning, intratumoral and peritumoral radiomics features, and clinical characteristics—demonstrated good discriminative performance, improved calibration, and potential clinical benefit. It may serve as an effective tool for distinguishing stage I from stage II–IV EC within the updated FIGO framework and provide imaging-based evidence to support preoperative staging and management.

## Supplementary information

ELECTRONIC SUPPLEMENTARY MATERIAL

## Data Availability

The datasets generated and/or analyzed during the current study are not publicly available due to institutional policy and patient privacy, but are available from the corresponding author on reasonable request.

## Abbreviations

ADC: Apparent diffusion coefficient
AFP: Alpha-fetoprotein
AUC: Area under the receiver operating characteristic curve
CA125: Cancer antigen 125
CA19-9: Cancer antigen 19–9
CEA: Carcinoembryonic antigen
CI: Confidence interval
DLRN: Deep-learning radiomics nomogram
DWI: Diffusion-weighted imaging
EC: Endometrial cancer
FIGO: International Federation of Gynecology and Obstetrics
FS-T2WI: Fat-suppressed T2-weighted imaging
Grad-CAM: Gradient-weighted Class Activation Mapping
HE4: Human epididymal protein 4
ICC: Intraclass correlation coefficient
OOF: Out-of-fold
ROI: Region of interest
SHAP: Shapley Additive exPlanations
