# Supplementary material for: MRI-based deep-learning radiomics nomogram for differentiating FIGO 2023 stage I from stage II–IV endometrial cancer: a multicenter study
Source: Insights Imaging. 2026 Nov 6;17:284. doi: 10.1186/s13244-026-02428-9 (PMC13743664; doi:10.1186/s13244-026-02428-9)
Supplement: Supplementary file 1 [file 13244_2026_2428_MOESM1_ESM.pdf]

MRI-Based Deep Learning Radiomics Nomogram for Differentiating FIGO 2023 Stage I from Stage II–IV Endometrial Cancer: a multicenter study

ELECTRONIC SUPPLEMENTARY MATERIAL

CONTENTS

Section 1 2023 FIGO staging of endometrial cancer 2

Section 2 Center information and MRI parameters 4

Section 3 Radiomics Analysis 5

Section 4 Deep Learning Analysis 7

Tables 8

Figures 13

Section 1 2023 FIGO staging of endometrial cancer

Stage	Description
**Stage I**	Confined to uterine corpus and ovary
**IA**	Disease limited to the endometrium or non-aggressive histological type, with < 50% myometrial involvement, no focal LVSI, or good disease prognosis.
**IA1**	Non-aggressive histological type limited to an endometrial polyp or confined to endometrium.
**IA2**	Non-aggressive histological type with < 50% myometrial involvement with no to focal LVSI.
**IA3**	Low-grade endometrioid carcinomas limited to uterus or ovary.
**IB**	Non-aggressive histological type with > 50% myometrial invasion, and no to focal LVSI.
**IC**	Aggressive histological subtype limited to a polyp or confined to endometrium.
**Stage II**	Invasion of cervical stroma with no extrauterine extension, or substantial LVSI, or aggressive histological type with any myometrial invasion.
**IIA**	Non-aggressive histological type with invasion of cervical stroma.
**IIB**	Non-aggressive histological type with substantial LVSI.
**IIC**	Aggressive histological subtype with any myometrial involvement.
**Stage III**	Local and/or regional metastasis of any histological subtype.
**IIIA**	Invasion of uterine serosa, adnexa, or both.
**IIIA1**	Spread to ovary or fallopian tube (excluding lesions that meet stage IA3 criteria).
**IIIA2**	Spread to uterine subserosa or through uterine serosa.
**IIIB**	Metastasis or direct spread to the vaginal canal and/or parametria or pelvic peritoneum.
**IIIB1**	Metastasis or direct spread to the vaginal canal and/or parametria.
**IIIB2**	Metastasis to pelvic peritoneum.
**IIIC**	Metastasis to pelvic and/or para-aortic lymph nodes.
**IIIC1**	Metastasis to pelvic lymph nodes.
**IIIC1i**	Micrometastasis to pelvic lymph nodes.
**IIIC1ii**	Macrometastasis to pelvic lymph nodes.
**IIIC2**	Metastasis to para-aortic lymph nodes up to the renal vessels, and/or pelvic lymph node metastasis.
**IIIC2i**	Micrometastasis to para-aortic lymph nodes up to the renal vessels, and/or pelvic lymph node metastasis.
**IIIC2ii**	Macrometastasis to para-aortic lymph nodes up to the renal vessels, and/or pelvic lymph node metastasis.
**Stage IV**	Spread to the bladder mucosa and/or intestinal mucosa and/or distant metastasis.

Stage	Description
**IVA**	Spread to the bladder mucosa and/or intestinal mucosa.
**IVB**	Abdominal peritoneal metastasis beyond the pelvic peritoneum.
**IVC**	Distant metastasis, including metastasis to any extra-abdominal or intra-abdominal lymph nodes superior to the renal vessels, lungs, liver, brain, or bone.

Section 2 Center information and MRI parameters

The multicenter study was conducted jointly by four centers:

Center I: the Affiliated Suzhou Hospital of Nanjing Medical University (1.5/3.0 T, Aera/Skyra, SIEMENS).

Center II: the Affiliated Huaian No. 1 People's Hospital Hospital of Nanjing Medical University (3.0 T, Ingenia CX, Philips; 1.5/3.0 T, Aera/ Avanto/ Spectra/ Verio, SIEMENS)

Center III: the First Affiliated Hospital of Soochow University (1.5/3.0 T, Ingenia Ambition S/ Ingenia, Philips; 3.0 T, Signa HDxt, GE; 3.0 T, Skyra, SIEMENS)

Scanners and imaging parameters of fat-suppressed T2-weighted (FS-T2W) from different centers are presented in **Table S1**.

Section 3 Radiomics Analysis

Radiomics features were extracted from the intratumoral and peritumoral ROIs on each of the three MRI sequences (FS-T2WI, DWI, and ADC) using the open-source PyRadiomics package (version 3.1.0), following the Imaging Biomarker Standardization Initiative. For each "sequence–ROI" combination, 14 shapes, 18 first-order, and 75 texture features (107 in total) were computed from the original images. Additionally, 279 features were derived from the Laplacian-of-Gaussian filter, and 744 from the wavelet filter. Thus, 1,130 candidate features were obtained for each "sequence–ROI" combination, yielding a total of 6,780 features across three sequences and two ROIs. Features with an ICC < 0.80 were excluded.

To identify stable and non-redundant radiomics features, feature selection and model development were performed using the training set only. (1) Mann–Whitney U tests ($P < 0.05$) were applied for preliminary dimensionality reduction. (2) A random forest model was used to rank feature importance, and highly correlated feature pairs were removed based on Spearman correlation analysis ($|r| > 0.80$) to mitigate redundancy and multicollinearity. (3) The remaining features were standardized using Z-scores, and a 10-fold cross-validated least absolute shrinkage and selection operator (LASSOCV) was used to determine the optimal regularization strength. With this regularization parameter fixed, 100 bootstrap resamples of the training set were performed for stability selection; the selection frequency of each feature by LASSO was recorded, and features with a selection frequency ≥ 0.60 were retained to form the final stable feature set. (4) A classification model was subsequently trained using this stable feature set. To reduce the potential influence of class imbalance, SMOTEENN was incorporated into the training pipeline to

combine synthetic minority oversampling with edited nearest-neighbor cleaning. The SMOTEENN sampling strategy and the class weights of the support vector machine were optimized during grid search. A support vector machine with an RBF kernel served as the classifier, and hyperparameters were tuned using repeated stratified cross-validation. The final model output out-of-fold (OOF) predicted probabilities for subsequent evaluation and fusion analyses. To facilitate fusion modeling, radiomics probabilities for the training set were obtained via OOF inference as described below.

In addition, to evaluate whether incorporating peritumoral radiomics information could further improve diagnostic performance, we constructed an original-ROI radiomics model for comparison. This model used features extracted only from the original ROI and followed the same feature selection, model training, and evaluation procedures as the combined model to ensure a fair comparison.

Section 4 Deep Learning Analysis

We developed a three-channel 3D deep learning classification model based on multiparametric MRI (FS-T2WI, DWI, and ADC). For each modality, a cubic patch centered on the lesion ROI was extracted using a bounding box covering the maximal tumor extent, and the patch was resampled to 96 × 96 × 96 voxels. Data augmentation, such as random flipping, 90° rotations, affine transformations/scaling, and intensity perturbations, was applied only to the training set to improve generalization. A 3D ResNet-18 backbone was used, and the model was optimized using binary cross-entropy loss with logits, with class imbalance addressed via a positive class weight. The model was optimized using AdamW (learning rate, 2×10^{-4}; weight decay, 1×10^{-4}). During training, early stopping was used: training was stopped after 10 consecutive epochs without improvement, and the model weights with the best validation AUC were retained (maximum 100 epochs; batch size = 16). Out-of-fold inference was performed on the training set according to a predefined fold table. Model training was performed using the open-source PyTorch framework (version 2.9.1, CUDA 12.8) on an NVIDIA GeForce RTX 4090 GPU.

Tables

Tabel S1. Different Scanners and Imaging Parameters of FS-T2W.

Centers	Manufacturer	Type	Sequence	Magnetic Field (T)	TR (ms)	TE (ms)	Slice Thickness (mm)	Matrix Size
**Center I**	SIEMENS	Skyra	SE	3.0	3020	101	6~9	320×320
	SIEMENS	Aera	SE	1.5	5250	85	6	320×320
**Center II**	Philips	Ingenia CX	SE	3.0	4935	80	4~5	480×480
	SIEMENS	Aera	SE	1.5	4410~5140	86~87	5~6	640×640
	SIEMENS	Avanto	SE	1.5	1100~5420	96~120	5~8	384×512
	SIEMENS	Spectra	SE	3.0	4500	96	4.5~5.5	640×640
	SIEMENS	Verio	SE	3.0	1700~5000	97~98	5~7	320×320/240×320
**Center III**	Philips	Ingenia Ambition S	SE	1.5	3333~4843	85~90	5~6.5	384×384/512×512
	Philips	Ingenia	SE	3.0	3385~4776	94~12	5~6	576×576/640×640
	GE	Signa HDxt	SE	3.0	4120	104	6	512×512
	SIEMENS	Skyra	SE	3.0	5000	77	5~6	640×640

FS-T2W, fat-suppressed T2-weighted; TR, repetition time; TE, echo time

Tabel S2. Slice thickness of MRI sequences across the three centers.

Parameter	Center 1	Center 2	Center 3
FS-T2WI slice thickness	6-9 mm	4-8 mm	5-6.5 mm
DWI slice thickness	5-8 mm	4-6.5 mm	4-8 mm
ADC slice thickness	derived from DWI	derived from DWI	derived from DWI

Tabel S3. Predictive performance of intratumoral, peritumoral, and combined intratumoral–peritumoral radiomics models.

Models	Internal test				External test			
	AUC (95% CI)	Acc	Sens	Spec	AUC (95% CI)	Acc	Sens	Spec
Intratumoral Radiomics	0.770 (0.689–0.850)	0.734	0.639	0.762	0.725 (0.656–0.757)	0.690	0.657	0.703
Peritumoral Radiomics	0.769 (0.667–0.871)	0.797	0.611	0.852	0.720 (0.648–0.793)	0.707	0.552	0.770
Combined Radiomics	0.793 (0.713–0.873)	0.734	0.722	0.738	0.743 (0.673–0.812)	0.659	0.687	0.648

Tabel S4. Sensitivity analysis of different deep learning patch definitions on the external test set.

DL input strategy	Test set	AUC	Accuracy	Sensitivity	Specificity
Original lesion-centered patch	Internal test	0.849	0.747	0.722	0.754
Enlarged peritumoral patch	Internal test	0.813	0.696	0.806	0.664
Original lesion-centered patch	External test	0.800	0.746	0.716	0.758
Enlarged peritumoral patch	External test	0.738	0.694	0.522	0.764

Tabel S5. Performance comparison of different deep learning backbones.

Models	Internal test				External test			
	**AUC (95% CI)**	**Acc**	**Sens**	**Spec**	**AUC (95% CI)**	**Acc**	**Sens**	**Spec**
3D ResNet-10	0.816 (0.739–0.892)	0.741	0.750	0.738	0.761 (0.691–0.832)	0.681	0.701	0.673
3D ResNet-18	0.849 (0.785–0.914)	0.747	0.722	0.754	0.800 (0.734–0.866)	0.746	0.716	0.758
3D ResNet-34	0.828 (0.757–0.899)	0.785	0.583	0.844	0.759 (0.688–0.830)	0.754	0.493	0.861
3D ResNet-50	0.824 (0.750–0.899)	0.797	0.528	0.877	0.733 (0.661– 0.806)	0.733	0.388	0.873
3D DenseNet-121	0.848 (0.777–0.920)	0.684	0.889	0.623	0.753 (0.681–0.825)	0.629	0.731	0.588
3D EfficientNet-B0	0.795 (0.712–0.879)	0.620	0.889	0.541	0.707 (0.631–0.783)	0.638	0.701	0.612

Figures

Supplementary Figure S1:

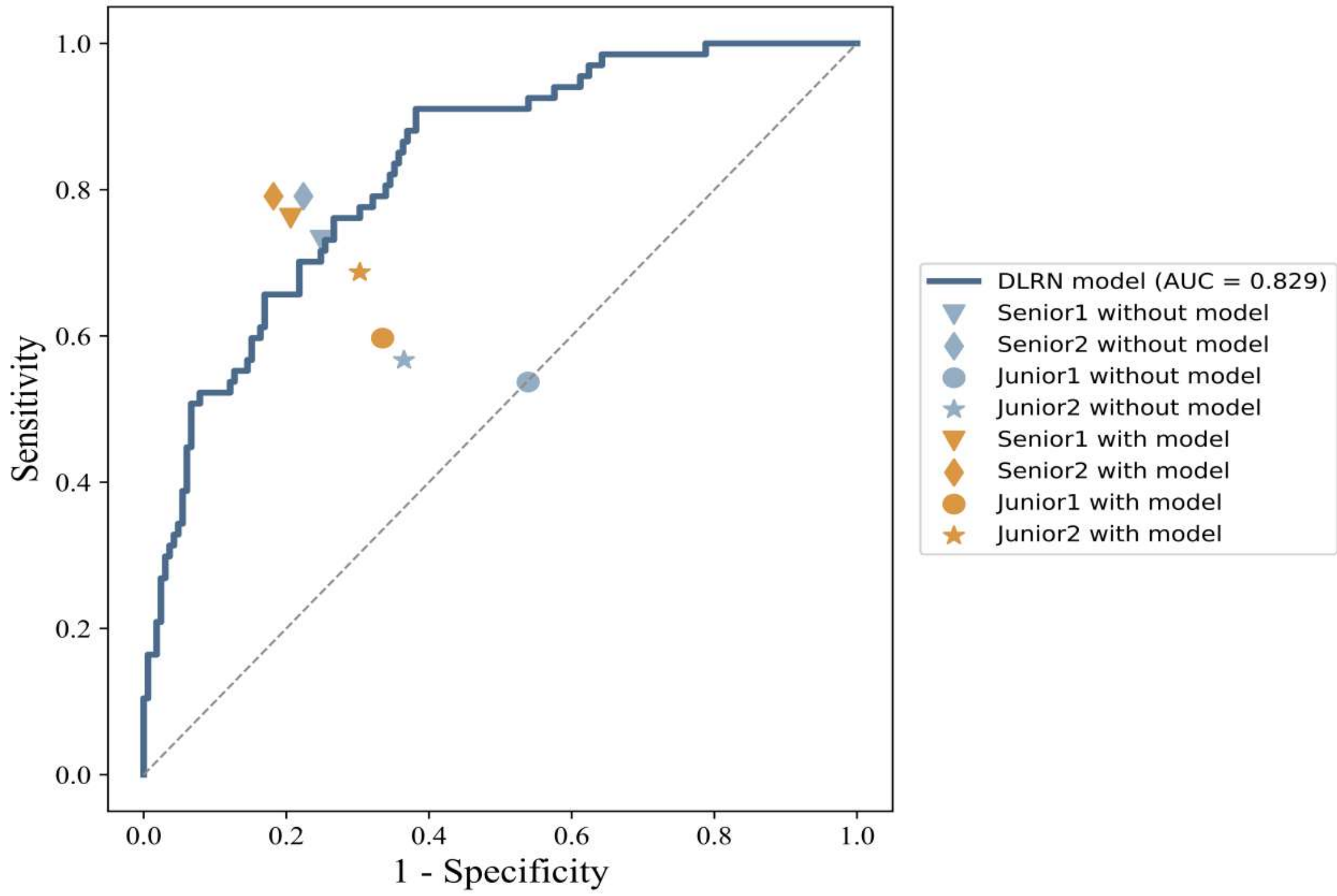

Supplementary Figure S1. Reader-performance analysis with and without DLRN assistance in the external test set. The ROC curve represents the DLRN model, and scatter points represent radiologists' performance before and after DLRN assistance.
